# Associations between socioprovincial factors and self-reported mental disorders among students from grade 4 to 8 in rural China

**DOI:** 10.1186/s13690-021-00580-w

**Published:** 2021-04-23

**Authors:** Ming Guan

**Affiliations:** 1 International Issues Center, Xuchang University, Xuchang, Road Bayi 88, Xuchang, Henan China; 2 Family Issues Center, Xuchang University, Road Bayi 88, Xuchang, Henan China; 3 School of Business, Xuchang University, Road Bayi 88, Xuchang, Henan China

**Keywords:** Socioprovincial factors, Self-reported mental disorders, Students from grade 4 to 8, Number of self-reported mental disorders, Rural China

## Abstract

**Background:**

The focus on child mental health in developing countries was increasing. However, little was known in China. This study aimed to explore the associations between socioprovincial factors and self-reported mental disorders in rural China.

**Methods:**

Data were from a publicly available survey with 54,498 students from Grade 4 to 8 in rural China. Chi-square test was used for descriptive analysis. Self-reported mental disorders included overall mental disorder, study anxiety, personal anxiety, loneliness, guilt, sensitivity, symptomatic psychosis, phobia, and impulsivity. Multiple logistic regressions and errors-in-variables regression models were employed to explore the associations between socioprovincial factors and mental disorders. Poisson regressions and errors-in-variables regression models were adopted to reveal the associations between socioprovincial factors and number of self-reported mental disorders.

**Results:**

Descriptive statistics showed that mental health was poor in rural adolescents in China. Logistic regression showed that the odds of overall mental disorder and study anxiety were 189% (AOR = 2.89, 95%CI: 2.76, 3.02) and 92% (OR = 1.92, 95%CI: 1.84, 2.00) in Gansu more than those in Anhui, while the odds of personal anxiety, guilt, sensitivity, symptomatic psychosis, and phobia were 92% (AOR = 0.08, 95%CI: 0.08, 0.09), 71% (AOR = 0.29, 95%CI: 0.27, 0.30), 88% (AOR = 0.12, 95%CI: 0.11, 0.13), 69% (AOR = 0.31, 95%CI: 0.29, 0.32), and 78% (AOR = 0.22, 95%CI: 0.21, 0.23) in Gansu less than those in Anhui. Moreover, Gansu (Poisson regression: IRR =1.45, 95%CI: 1.42–1.47; errors-in-variables regression: Coefficient = 0.26, 95%CI: 0.16, 0.36), Ningxia (Poisson regression: IRR =1.63, 95%CI: 1.60–1.67; errors-in-variables regression: Coefficient = 0.43, 95%CI: 0.32, 0.53), Qinghai (Poisson regression: IRR =1.65, 95%CI: 1.60–1.69; errors-in-variables regression: Coefficient = 0.44, 95%CI: 0.34, 0.55), and Shaanxi (Poisson regression: IRR =1.28, 95%CI: 1.25–1.30; errors-in-variables regression: Coefficient = 0.11, 95%CI: 0.00, 0.21) were significantly associated with the number of self-reported mental disorders.

**Conclusion:**

Class and provincial disparities in self-reported mental disorders were reported among the students from Grade 4 to 8 in rural China. Mental health care supported by governments and schools could be an effective way to reduce the disparities in mental disorders among the adolescents.

## Background

There was a growing body of evidence suggesting that adolescents were susceptible to a variety of mental disorders among adolescents. Currently, mental disorders including anxiety disorders [1] and depressive symptoms [2] in adolescents had been attracted by academic circle. For example, an epidemiological study found a high incidence of comorbidity in children and adolescent psychiatry [3]. Another study indicated that depressive and anxiety symptoms often co-occurred [4]. Empirically, early adolescent anxiety disorders were related to lower self-esteem from early adolescence through young adulthood, with social phobia having the greatest impact [5].

Regarding socio-geographical factors, prior research reported that socioregional factors [6], social gradient in antisocial behavior [7], teacher perspectives [8], and income change [9] had associations with mental disorders among adolescents. Furthermore, a study reported race moderated the link between physical discipline and externalizing behavior problems for European American adolescents during Grades 6 and 8 [10]. Another large epidemiological study in Israel found an association between religion/ethnicity and internalizing and externalizing disorders in Muslim adolescents [11].

Multiple studies documented the associations between household wealth and mental health changes among adolescents worldwide. For instance, a study concluded that national wealth had associations with poor mental health at the aggregated level [12]. Regarding the association between wealth inequality and the risk of mental disability in the Chinese population, a study with nationally represented, population-based data from the second China National Sample Survey on Disability 2006 suggested that wealth was a significant predictor in the distribution of mental disability [13]. Furthermore, a study with data from 2060 young adults aged 18–27 in 2005–2011 from the Panel Study of Income Dynamics suggested family wealth affected mental health [14]. Likewise, a study with data (2001–2012) from the Household, Income and Labour Dynamics in Australia survey reported that low household wealth prior to disability acquisition in adulthood resulted in a greater deterioration in mental health than those with high wealth [15]. Additionally, a study with the case of three distinctive communities in Haiti found household agricultural wealth was significantly and strongly associated with both reductions in depression symptoms and anxiety symptoms [16].

However, there were a limited number of studies to report the associations between socioprovincial factors and self-reported mental disorders in rural China. Prior research on the associations of socioprovincial factors with mental health were seldom documented because the data employed did not include provincial variable. This study tried to fill into the gaps.

## Methods

### Data source

This study employed a publicly available survey data [17]. The dataset was aggregated from 10 different school-level surveys that conducted in rural areas of five provinces of from 2008 to 2015, which covered 54,498 students from Grade 4 to 8 across 65 counties in rural China. The dataset included information on student characteristics (gender, grade, and surveyed provinces) and family background (household asset) as well as mental health situations.

In order to measure psychological test of well-being, this data employed Mental Health Test (MHT) which was adapted by Professor Zhou Bucheng of East China Normal University from the General Anxiety Test developed by Kiyoshi Suzuki in Japan as an internationally standardized test for anxiety in children. MHT was a 100-item self-report inventory, in which consisted of 10-item overall mental disorder (82, 84, 86, 88, 90, 92, 94, 96, 98, and 100), 15-item study anxiety (1, 2, 3, 4, 5, 6, 7, 8, 9, 10, 11, 12, 13, 14, and 15), 10-item personal anxiety (16, 17, 18, 19, 20, 21, 22, 23, 24, and 25), 10-item loneliness (26, 27, 28, 29, 30, 31, 32, 33, 34, and 35), 10-item guilty (36, 37, 38, 39, 40, 41, 42, 43, 44, and 45), 10-item sensitivity (46, 47, 48, 49, 50, 51, 52, 53, 54, and 55), 15-item symptomatic psychosis (56, 57, 58, 59, 60, 61, 62, 63, 64, 65, 66, 67, 68, 69, and 70), 10-item phobia (71, 72, 73, 74, 75, 76, 77, 78, 79, and 80), and 10-item impulsivity (81, 83, 85, 87, 89, 91, 93, 95, 97, and 99). The response options of each item were yes (=1) and no (=0). Thus, the maximum subscale scores of overall mental disorder, study anxiety, personal anxiety, loneliness, guilty, sensitivity, symptomatic psychosis, phobia, and impulsivity were 10 points, 15 points, 10 points, 10 points, 10 points, 10 points, 15 points, 10 points, and 10 points, respectively. Here, cut-off point of 7 was implemented in the subcategories of overall mental disorder, study anxiety, personal anxiety, loneliness, guilty, sensitivity, symptomatic psychosis, phobia, and impulsivity. Simultaneously, the eight subscales were divided by 7 and more points (yes = 1) less than 7 points and (no = 0). The key variables’ definition, scoring, and standardization were descripted in literature [18].

### Main variables

Here, dependent variables referred to overall mental disorder, mental subscales (study anxiety, personal anxiety, loneliness, guilt, sensitivity, symptomatic psychosis, phobia, and impulsivity), and number of self-reported mental disorders. Additionally, overall mental disorder and mental subscales were reflected by the questions: “Percentage of kids who have 7+ points in any category” and “Whether or not kids have 7+ points in study anxiety, personal anxiety, lonely, guilty, sensitive, symptomatic, phobic, or impulsive subcategory”, respectively. Their response options were yes (=1) and no (=0). Number of self-reported mental disorders was calculated by arithmetic sum of response options of study anxiety, personal anxiety, loneliness, guilt, sensitivity, symptomatic psychosis, phobia, and impulsivity.

Independent variables included school grade (4, 5, 7, and 8), gender (female = 0, male = 1), surveyed provinces (Anhui, Gansu, Ningxia, Qinghai, and Shaanxi), and standardized index for household wealth. Standardized index for household wealth was constructed by polychoric principal components analysis with dichotomous household items, such as a microwave, refrigerator, fan, etc.

### Statistical analysis

The associations between socioprovincial factors and self-reported mental disorders were mainly calculated by multiple logistic regressions. Due to response options with rare “yes” relative to “no” in loneliness and impulsivity, the associations between socioprovincial factors and mental disorders were calculated by complementary log-log regression (stata program: cloglog). The associations between socioprovincial factors and number of self-reported mental disorders were explored by Poisson regression with incidence-rate ratios (IRR).

Due to survey data, measurement errors could be considered in the errors-in-variables regression models when the subjective response variables were possibly measured with errors. Here, the assumed reliability of standardized index for household wealth was defined as 0.85. Subsequently, errors-in-variables regressions on associations between socioprovincial factors and mental disorders were conducted.

## Results

### Descriptive statistics

Among the available 54,498 participants, males accounted for 52.04%, and females accounted for 47.96%. Regarding surveyed provinces, the majority of the sample was from Shaanxi (49.50%), followed by Gansu (26.30%), Ningxia (14.64%), Qinghai (6.88%), and Anhui (2.68%). Regarding school grades, the majority of the sample were in Grade 4 (47.22%), followed by Grade 5 (33.07%), Grade 8 (10.09%), and Grade 7 (9.63%). Regarding mental morbidity, the number of self-reported mental disorders was distributed as 0 (29.42%), 1 (33.89%), 2 (20.12%), 3 (9.20%), 4 (4.38%), 5 (1.95%), 6 (0.79%), 7 (0.22%), and 8 (0.02%). In Table 1, there were significant gender differences in the case of surveyed provinces, school grade, overall mental disorder, study anxiety, personal anxiety, loneliness, guilt, sensitivity, symptomatic psychosis, phobia, and impulsivity.

**Table 1:** Sample characteristics by gender

	Female		Male		Chi-square	*P* value
	N	%	N	%		
Surveyed provinces (*N* = 54,498)					35.5147	0.000***
Anhui	715	1.31	746	1.37		
Gansu	7098	13.02	7234	13.27		
Ningxia	3924	7.20	4057	7.44		
Qinghai	1793	3.29	1956	3.59		
Shaanxi	12,607	23.13	14,368	26.36		
School Grade (*N* = 54,498)					14.4172	0.002***
4	12,314	22.60	13,420	24.62		
5	8811	16.17	9209	16.90		
7	2428	4.46	2819	5.17		
8	2584	4.74	2913	5.35		
Standardized index for household wealth (median, Interquartile Range) (*N* = 48,956)	−.2809, (−.6637, .8124)		−.2611, (−.6459, .8700)			
Mental disorder (*N* = 54,498)					268.4976	0.000***
No	6819	12.51	9215	16.91		
Yes	19,318	35.45	19,146	35.13		
Study anxiety (*N* = 54,498)					205.0397	0.000***
No	9212	16.90	11,689	21.45		
Yes	16,925	31.06	16,672	30.59		
Personal anxiety (*N* = 54,498)					60.9713	0.000***
No	24,169	44.35	26,699	48.99		
Yes	1968	3.61	1662	3.05		
Loneliness (*N* = 54,498)					1.7715	0.183
No	25,640	47.05	27,865	51.13		
Yes	497	0.91	496	0.91		
Guilt (*N* = 54,498)					342.9068	0.000***
No	20,522	37.66	24,009	44.05		
Yes	5615	10.30	4352	7.99		
Sensitivity (*N* = 54,498)					3.9022	0.048**
No	22,928	42.07	25,035	45.94		
Yes	3209	5.89	3326	6.10		
Symptomatic psychosis (*N* = 54,498)					14.6444	0.000***
No	20,441	37.51	22,560	41.40		
Yes	5696	10.45	5801	10.64		
Phobia (*N* = 54,498)					1.4e+ 03	0.000***
No	21,679	39.78	26,488	48.60		
Yes	4458	8.18	1873	3.44		
Impulsivity (*N* = 54,498)					2.3446	0.126
No	25,545	46.87	27,662	50.76		
Yes	592	1.09	699	1.28		

Note: *p < 0.1, **p < 0.05, ***p < 0.01

### Associations between socioprovincial factors and mental disorders

Regarding gender, in Tables 2 and 3, significant adjusted odds ratios (AOR) and significant coefficients of male indicated male students were less susceptible to mental disorders than female students. Considering school grade, in Tables 2 and 3, significant adjusted odds ratios and significant coefficients indicated students in Grade 5 were less susceptible to mental disorders than students in Grade 4. In addition, students in Grades 7 and 8 were more susceptible to mental disorders than students in Grade 4.

**Table 2:** Logistic and linear regression on associations between socioprovincial factors and mental disorders. AOR (95%CI), Coefficient (95%CI)

	OMD	Study anxiety	Personal anxiety	Loneliness	Guilt	Sensitivity	Symptomatic psychosis	Phobia	Impulsivity
Gender									
Female	Reference	Reference	Reference	Reference	Reference	Reference	Reference	Reference	Reference
Male	0.75*** (0.72,0.78)	0.77*** (0.75,0.80)	0.71*** (0.67,0.77)	− 0.09 (− 0.22,0.04)	0.62***(0.60,0.65)	0.86***(0.81,0.91)	0.87***(0.84,0.91)	0.32***(0.30,0.34)	0.05 (− 0.06, 0.17)
Grade									
4	Reference	Reference	Reference	Reference	Reference	Reference	Reference	Reference	Reference
5	0.99 (0.95,1.04)	1.01 (0.97,1.05)	0.90** (0.83,0.98)	− 0.23***(− 0.39,-0.08)	0.93***(0.88,0.97)	0.87***(0.81,0.92)	0.78***(0.74,0.81)	0.81***(0.76,0.86)	−0.03(− 0.17,0.11)
7	1.54*** (1.43,1.67)	1.51*** (1.41,1.63)	1.37*** (1.19,1.58)	0.39***(0.13,0.64)	1.13***(1.03,1.24)	1.26***(1.13,1.39)	1.26***(1.15,1.38)	0.91 (0.81,1.03)	0.74***(0.53,0.96)
8	1.74*** (1.61,1.88)	1.65*** (1.54,1.78)	1.57*** (1.38,1.80)	0.60***(0.36,0.84)	1.15***(1.05,1.26)	1.70***(1.54,1.87)	1.42***(1.31,1.55)	0.93 (0.83,1.04)	0.87***(0.66,1.07)
Surveyed provinces									
Anhui	Reference	Reference	Reference	Reference	Reference	Reference	Reference	Reference	Reference
Gansu	2.89*** (2.76,3.02)	1.92*** (1.84,2.00)	0.08*** (0.08,0.09)	0.71* (−0.02,1.44)	0.29***(0.27,0.30)	0.12***(0.11,0.13)	0.31***(0.29,0.32)	0.22***(0.21,0.23)	0.37***(−0.21,0.94)
Ningxia	3.55*** (3.35,3.76)	2.15*** (2.04,2.27)	0.10*** (0.09,0.11)	0.80** (0.06,1.54)	0.30***(0.29,0.32)	0.14***(0.13,0.15)	0.43***(0.41,0.46)	0.30***(0.28,0.32)	0.75 (0.18,1.33)
Qinghai	4.45*** (4.09,4.84)	2.43*** (2.26,2.62)	0.09*** (0.08,0.10)	1.16*** (0.41,1.90)	0.26***(0.23,0.28)	0.12***(0.11,0.13)	0.54***(0.50,0.59)	0.26***(0.23,0.29)	1.11*** (0.53,1.69)
Shaanxi	1.92*** (1.83,2.00)	1.31*** (1.26,1.37)	0.07*** (0.07,0.08)	0.47(−0.27,1.20)	0.26***(0.25,0.27)	0.15***(0.14,0.16)	0.22***(0.21,0.23)	0.20***(0.19,0.22)	0.33(−0.24,0.91)
SIHW	1.00*** (1.00,1.00)	1.00** (1.00,1.00)	1.00*** (1.00,1.00)	0.00(−0.00,0.00)	1.00***(1.00,1.00)	1.00***(1.00,1.00)	1.00***(1.00,1.00)	1.00***(1.00,1.00)	0.00** (0.00,0.00)
Constant				−4.62***(−5.35,-3.88)					−4.43***(−5.00, −3.85)
N	48,956	48,956	48,956	48,956	48,956	48,956	48,956	48,956	48,956

Note: *p < 0.1, **p < 0.05, ***p < 0.01. *SIHW* Standardized index for household wealth. *OMD* Overall mental disorder

**Table 3:** Errors-in-variables regression on associations between socioprovincial factors and mental disorders, Coefficient (95%CI)

	OMD	Study anxiety	Personal anxiety	Loneliness	Guilt	Sensitivity	Symptomatic psychosis	Phobia	Impulsivity
Gender									
Female	Reference	Reference	Reference	Reference	Reference	Reference	Reference	Reference	Reference
Male	−0.06*** (−0.07, −0.06)	−0.06*** (− 0.07, − 0.05)	−0.02*** (− 0.02, − 0.01)	−0.00(− 0.00, 0.00)	−0.06***(− 0.07, − 0.06)	−0.01**(− 0.01, − 0.00)	−0.01*** (− 0.02, − 0.01)	−0.11***(− 0.11, − 0.10)	0.00(− 0.00, 0.00)
Grade									
4	Reference	Reference	Reference	Reference	Reference	Reference	Reference	Reference	Reference
5	−0.01*** (− 0.02, − 0.01)	0.00(− 0.01, 0.01)	0.00(− 0.00, 0.01)	−0.00*** (− 0.01, − 0.00)	0.01* (− 0.00, 0.02)	0.01*** (0.01, 0.02)	−0.02*** (− 0.03, − 0.01)	−0.00(− 0.01, 0.00)	−0.00(− 0.00, 0.00)
7	0.09*** (0.07, 0.11)	0.10*** (0.08, 0.12)	0.02*** (0.01, 0.03)	0.01*** (0.00, 0.01)	0.03*** (0.01, 0.04)	0.04*** (0.03, 0.05)	0.04*** (0.03, 0.06)	−0.00(− 0.01, 0.01)	0.02*** (0.01, 0.02)
8	0.11*** (0.10, 0.13)	0.12*** (0.10, 0.14)	0.03*** (0.02, 0.04)	0.01*** (0.01, 0.02)	0.03*** (0.01, 0.04)	0.08*** (0.07, 0.09)	0.06*** (0.05, 0.08)	−0.00(−0.01, 0.01)	0.02*** (0.02, 0.03)
Surveyed provinces									
Anhui	Reference	Reference	Reference	Reference	Reference	Reference	Reference	Reference	Reference
Gansu	0.10*** (0.06, 0.13)	0.14*** (0.10, 0.18)	0.02* (−0.00, 0.04)	0.01*(− 0.00, 0.02)	−0.01(− 0.04, 0.02)	0.01(− 0.02, 0.03)	0.07*** (0.04, 0.11)	0.02(− 0.00, 0.04)	0.01(− 0.00, 0.02)
Ningxia	0.14*** (0.10, 0.17)	0.17*** (0.13, 0.21)	0.03*** (0.01, 0.05)	0.01*(−0.00, 0.02)	− 0.01(− 0.04, 0.03)	0.02(− 0.01, 0.05)	0.14*** (0.10, 0.17)	0.05***(0.03, 0.08)	0.02*** (0.00, 0.03)
Qinghai	0.18***(0.14, 0.22)	0.19*** (0.15, 0.23)	0.02** (0.00, 0.04)	0.02 (0.01, 0.03)	−0.03* (− 0.06, 0.00)	0.00(− 0.03, 0.03)	0.18*** (0.14, 0.21)	0.03**(0.01, 0.06)	0.03*** (0.01, 0.04)
Shaanxi	0.01(−0.03, 0.04)	0.05**(0.01, 0.09)	0.01(−0.01, 0.03)	0.01(−0.01, 0.02)	− 0.03* (− 0.06, 0.00)	0.02*(− 0.00, 0.05)	0.03(− 0.01, 0.06)	0.01(− 0.01, 0.04)	0.01(−0.00, 0.02)
SIHW	0.00(−0.00, 0.00)	0.00(−0.00, 0.00)	− 0.00(− 0.00, 0.00)	0.00(− 0.00, 0.00)	−0.00(− 0.00, 0.00)	0.00(− 0.00, 0.00)	0.00(− 0.00, 0.00)	0.00(− 0.00, 0.00)	0.00**(0.00, 0.00)
Constant	0.65*** (0.62, 0.69)	0.52*** (0.48, 0.56)	0.05*** (0.03, 0.07)	0.01**(0.00, 0.02)	0.22*** (0.19, 0.25)	0.09*** (0.06, 0.11)	0.15*** (0.11, 0.18)	0.15***(0.12, 0.17)	0.01(−0.00, 0.02)
R-squared	0.0200	0.0158	0.0032	0.0014	0.0075	0.0075	0.0137	0.0297	0.0034
N	48,956	48,956	48,956	48,956	48,956	48,956	48,956	48,956	48,956

Note: *p < 0.1, **p < 0.05, ***p < 0.01. *SIHW* Standardized index for household wealth. *OMD* Overall mental disorder

Regarding surveyed provinces in Table 2, the odds of overall mental disorder and study anxiety were 189% (AOR = 2.89, 95%CI: 2.76, 3.02) and 92% (OR = 1.92, 95%CI: 1.84, 2.00) in Gansu more than those in Anhui, respectively, while the odds of personal anxiety, guilt, sensitivity, symptomatic psychosis, and phobia were 92% (AOR = 0.08, 95%CI: 0.08, 0.09), 71% (AOR = 0.29, 95%CI: 0.27, 0.30), 88% (AOR = 0.12, 95%CI: 0.11, 0.13), 69% (AOR = 0.31, 95%CI: 0.29, 0.32), and 78% (AOR = 0.22, 95%CI: 0.21, 0.23) in Gansu less than those in Anhui, respectively. Similarly, the odds of overall mental disorder and study anxiety were 255% (AOR = 3.55, 95%CI: 3.35, 3.76) and 115% (AOR = 2.15, 95%CI: 2.04, 2.27) in Ningxia more than those in Anhui, respectively, while the odds of personal anxiety, guilt, sensitivity, symptomatic psychosis, and phobia were 90% (AOR = 0.10, 95%CI: 0.09, 0.11), 70% (AOR = 0.30, 95%CI: 0.29, 0.32), 86% (AOR = 0.14, 95%CI: 0.13, 0.15), 57% (AOR = 0.43, 95%CI: 0.41, 0.46), and 70% (AOR = 0.30, 95%CI: 0.28, 0.32) in Ningxia less than those in Anhui, respectively. Likewise, the odds of overall mental disorder and study anxiety were 345% (AOR = 4.45, 95%CI: 4.09, 4.84) and 143% (AOR = 2.43, 95%CI: 2.26, 2.62) in Qinghai more than those in Anhui, respectively, while the odds of personal anxiety, guilt, sensitivity, symptomatic psychosis, and phobia were 91% (AOR = 0.09, 95%CI: 0.08, 0.10), 74% (AOR = 0.26, 95%CI: 0.23, 0.28), 88% (AOR = 0.12, 95%CI: 0.11, 0.13), 46% (AOR = 0.54, 95%CI: 0.50, 0.59), and 74% (AOR = 0.26, 95%CI: 0.23, 0.29) in Qinghai less than those in Anhui, respectively. Additionally, the odds of overall mental disorder and study anxiety were 92% (AOR = 1.92, 95%CI: 1.83, 2.00) and 31% (AOR = 1.31, 95%CI: 1.26, 1.37) in Shaanxi more than those in Anhui, respectively, while the odds of personal anxiety, guilt, sensitivity, symptomatic psychosis, and phobia were 93% (AOR = 0.07, 95%CI: 0.07, 0.08), 74% (AOR = 0.26, 95%CI: 0.25, 0.27), 85% (AOR = 0.15, 95%CI: 0.14, 0.16), 78% (AOR = 0.22, 95%CI: 0.21, 0.23), and 80% (AOR = 0.20, 95%CI: 0.19, 0.22) in Shaanxi less than those in Anhui, respectively. Significantly positive and negative coefficients in Table 3 basically reflected the similar associations.

Simultaneously, in Tables 2 and 3, significant adjusted odds ratios and significant coefficients indicated standardized index for household wealth had no associations with mental disorders.

### Associations between socioprovincial factors and number of self-reported mental disorders

In Table 4, male was significantly associated with the number of self-reported mental disorders (Poisson regression: IRR = 0.82, 95%CI: 0.81–0.84; errors-in-variables regression: Coefficient = − 0.27, 95%CI: − 0.29, − 0.24). Regarding school grade, Grades 7 (Poisson regression: IRR =1.22, 95%CI: 1.18–1.26; errors-in-variables regression: Coefficient = 0.25, 95%CI: 0.20, 0.30) and 8 (Poisson regression: IRR =1.31, 95%CI: 1.27–1.35; errors-in-variables regression: Coefficient = 0.36, 95%CI: 0.31, 0.40) were significantly associated with the number of self-reported mental disorders. Regarding surveyed provinces, Gansu (Poisson regression: IRR =1.45, 95%CI: 1.42–1.47; errors-in-variables regression: Coefficient = 0.26, 95%CI: 0.16, 0.36), Ningxia (Poisson regression: IRR =1.63, 95%CI: 1.60–1.67; errors-in-variables regression: Coefficient = 0.43, 95%CI: 0.32, 0.53), Qinghai (Poisson regression: IRR =1.65, 95%CI: 1.60–1.69; errors-in-variables regression: Coefficient = 0.44, 95%CI: 0.34, 0.55), and Shaanxi (Poisson regression: IRR =1.28, 95%CI: 1.25–1.30; errors-in-variables regression: Coefficient = 0.11, 95%CI: 0.00, 0.21) were significantly associated with the number of self-reported mental disorders. Also, standardized index for household wealth was significantly associated with the number of self-reported mental disorders (Poisson regression: IRR =1.00, 95%CI: 1.00–1.00).

**Table 4:** Associations between socioeconomic factors and number of mental disorders. IRR (95%CI), Coefficient (95%CI)

	Poisson regression		Errors-in-variables regression	
	IRR	95%CI	Coefficient	95%CI
Gender				
Female	Reference		Reference	
Male	0.82***	0.81, 0.84	−0.27***	−0.29, −0.24
School Grade				
4	Reference		Reference	
5	1.01	0.99, 1.03	−0.00	−0.03, 0.02
7	1.22***	1.18, 1.26	0.25***	0.20, 0.30
8	1.31***	1.27, 1.35	0.36***	0.31, 0.40
Surveyed provinces				
Anhui	Reference		Reference	
Gansu	1.45***	1.42, 1.47	0.26***	0.16, 0.36
Ningxia	1.63***	1.60, 1.67	0.43***	0.32, 0.53
Qinghai	1.65***	1.60, 1.69	0.44***	0.34, 0.55
Shaanxi	1.28***	1.25, 1.30	0.11**	0.00, 0.21
Standardized index for household wealth	1.00***	1.00, 1.00	0.00	−0.00, 0.00
Constant			1.20***	1.10, 1.30
Log likelihood	−75,234.409			
R-squared			0.0212	
N	48,956		48,956	

Note: *p < 0.1, **p < 0.05, ***p < 0.01. *SIHW* Standardized index for household wealth

## Discussion

Notably, high prevalence of self-reported mental disorders was reported among the sampled students. The associations between socioprovincial factors and self-reported mental disorders and the number of self-reported mental disorders were confirmed. Besides gender disparities in mental disorders, there existed class and provincial disparities in mental disorders among the sample.

Regarding gender, this study was consistent with a series of early studies. For example, a Jordan adolescents study reported significant associations of age and gender with high prevalence of mental disorders [19]. A study with data from China National Sample Surveys on Disability in 1987 and 2006 indicated that gender of children was consistently associated with psychiatric disability among children [20]. The finding in this study was also in line with a study in South Korea which reported adolescent depression experience was highly associated with gender mix in the school [21]. In this study, there might be gender disparities between mental disorders.

Regarding schooling, this study was in line with a series of early studies. For example, a study demonstrated important mental health issues with a high incidence of comorbidities in poverty-stricken areas and left-behind children and adolescents in 40 primary and middle schools [22]. Globally, a substantial study also documented high mental disorders prevalence among adolescent students in Brazilian [23], left behind adolescents in Anhui province [24], adolescent age groups in Saudi Arabia [25], Australian child and adolescents [26], Hungarian children [27], and Lithuanian youth [28]. Additionally, an available research on the prevalence of child and adolescent psychiatric disorders in India was reviewed, synthesized, and evaluated [29]. The novel finding in this study was there existed class disparities in mental disorders among the students. Meanwhile, the students in higher class were more likely to be susceptible to mental disorders than those in lower class in junior school. The students in elementary school were less likely to be susceptible to mental disorders than those in junior school.

IRR of standardized index for household wealth was equal to 1, which indicated that household wealth was not the main determinant of the number of mental disorders. This finding could be verified by a current research which reported family functioning had associations with adolescent health and emotional well-being [30]. Furthermore, the research outcome was not in congruent with the published associations between wealth inequality and positive emotion [31, 32].

To the best of the knowledge of the author, this was the first study to report provincial disparities of mental disorders in rural China. Some early studies documented mental health of school students in Hong Kong, Shanghai, and Beijing [33], Shanghai [34–36], and Sichuan [22]. Likewise, mental health in rural China also was reported in northwestern China [37] and western China [38]. Compared with these studies, the novel finding in this study highlighted provincial disparities of mental disorders in rural China.

### Policy implications

This study highlighted the importance of socioprovincial factors in mental health. Adolescent mental disorders were confirmed to represent a risk marker for a number of later adverse outcomes [39]. Socioeconomic disadvantage and psychological deficits were found to contribute to criminal offending independently and with roughly equal magnitude [40]. Thus, adolescent psychopathology and support need be provided to minimize adverse outcomes. Regarding the provincial differences among ORs and IRRs, to my opinion, governments and schools should concern the differences of mental disorders between province-level units in China. Obviously, governments and schools were responsible for provincial imbalance of mental health service in rural China.

Understanding the associations between socioprovincial factors and self-reported mental disorders could help identify high-risk adolescents and take steps to minimize their disparities in mental disorders. Some similar studies recommended school mental health resources [41], developing personalized approaches to clinical assessment [42], adaptive interventions [43], and increasing health workforce [44] should be adopted to prevent and reduce the prevalence of mental disorders among the students. On the basis of the results in this study, optional prevention measures against mental orders need to smooth the difference of funds, human resource, and facilities among schools and provinces. Mental health care policies aimed at a redistribution of health resources at the provincial level also could decrease health service utilization inequalities in the adolescent mental well-beings.

### Limitations

Some limitations in this study should be highlighted. First, this study employed a second-handed survey data which did not provided specific items of MHT. Thus, reliability (split-half/Cronbach’s alpha, test-retest reliability, alternate form reliability, inter-rater reliability) and validity (content validity, construct validity, convergent or concurrent validity, discriminant validity, criterion (or predictive) validity) could not be calculated. Second, only five provinces were analyzed in this study. The research outcomes could be limitedly generalized to the whole country. Finally, several key definitions were ambiguous. Taking household wealth as an example, whether the wealth was parental or students’ was not defined. Meanwhile, a study with data from the UK Millennium Cohort Study indicated children’s mental health was not influenced by parental housing wealth but family permanent income and socioeconomic characteristics [45]. In addition, some key variables including socioeconomic inequalities [46], socioeconomic status [47], length of residence [48], unhealthy behaviours [49], and physiological engagement in social contexts [50], which were confirmed to be associated with adolescent mental disorders, were not included. Thus, mediating and moderating analyses could not be conducted. Accordingly, further research needs more variables to gain a better understanding of the disparities in student mental health.

## Conclusion

In conclusion, this study reflected the associations between socioprovincial factors and self-reported mental disorders among students from Grade 4 to 8 in rural China. Especially, this study reported disparities in gender, class, and province regarding mental disorders. Regarding the situation reflected by this study, public policy intervention should be highlighted and redesigned to solve the mental disorders of the adolescents in rural areas of China.

## Data Availability

https://dataverse.harvard.edu/dataverse/harvard?q=Rozelle%20Scott

## Abbreviations

MHT: Mental Health Test
IRR: Incidence-rate ratios
AOR: Adjusted odds ratio
CI: Confidence interval
SIHW: Standardized index for household wealth
OMD: Overall mental disorder
